# Ladies in waiting: the timeliness of first trimester services in New Zealand

**DOI:** 10.1186/1742-4755-7-19

**Published:** 2010-07-23

**Authors:** Martha Silva, Rob McNeill, Toni Ashton

**Affiliations:** 1 Senior Research Fellow, Health Systems, School of Population Health, University of Auckland, Private Bag 92019, Auckland 1142, New Zealand; 2 Lecturer, Health Systems, School of Population Health, University of Auckland, Private Bag 92019, Auckland 1142, New Zealand; 3 Associate Professor, Health Systems, School of Population Health, University of Auckland, Private Bag 92019, Auckland 1142, New Zealand

## Abstract

**Background:**

Termination of pregnancy (TOP) services are a core service in New Zealand. However, compared to other developed countries, TOP services are accessed significantly later in the first trimester, increasing the risk for complications. The aim of this study is to examine the timeliness of access to first trimester TOP services and establish the length of delay between different points in the care pathway for these services.

**Methodology:**

Data were collected from all patients attending nine TOP clinics around the country between February and May 2009 (N = 2950). Patient records were audited to determine the timeline between the first point of entry to the health system to the date of termination. In addition, women were invited to fill out a questionnaire to identify personal level factors affecting access to services (N = 1086, response rate = 36.8%).

**Results:**

Women waited an average of almost 25 days between the date of the first visit with the referring doctor and the date of their termination procedure. There was a delay of 10 days between the first visit with the referring doctor and the date that the appointment for the procedure was booked, and a further 10 days delay between the date the appointment was booked and the first appointment date. Over half of the women in this study had their pregnancy terminated at ten weeks or above.

**Conclusion:**

Women in New Zealand are subject to a lengthy delay while seeking TOP services. Efforts should be made by TOP clinics as well as referring doctors to reduce the waiting times for this service.

## Introduction

Termination of pregnancy (TOP) services are a core service in New Zealand, accessed by a large proportion of the female population. National statistics indicate that approximately one quarter of all pregnancies in the country end with a TOP, and one in every four women undergoes a TOP in her lifetime [1]. With an induced pregnancy termination rate of 19.7 per thousand women of reproductive age, New Zealand joins the United States and the United Kingdom among the countries with high induced pregnancy termination rates [1,2].

Although TOP is a safe procedure when conducted under hygienic conditions by a trained provider, the risk for clinical complications increases with gestational age [3-6]. Studies in the USA and Denmark found that abortion-related mortality increases exponentially with additional weeks of gestation, and women who have an induced abortion in their second trimester are significantly more likely to die of related causes compared to women who had the procedure before or at 8 weeks [3]. Given the evidence in favour of reducing the gestational age at termination, countries such as The United Kingdom have developed official guidelines that not only address clinical and technical issues, but also guide referral systems to help ensure the best clinical outcomes for their patients. The UK guidelines recommend that services offer arrangements to minimize delay in an attempt to minimize complications associated with later TOP procedures. To this end, they recommend that women requesting TOP ideally be offered an assessment appointment within 5 days of referral and no later than 2 weeks from referral, and be able to undergo a TOP procedure within 7 days and no longer than 2 weeks after the decision to proceed has been made. These guidelines state that no woman should wait longer than 3 weeks from her initial referral to the time of TOP [7].

In New Zealand, although 87.1% of TOPs are conducted during the first trimester, the average gestational age at termination is relatively late within the first trimester [8]. Currently it is unknown why TOP services are delivered relatively late in New Zealand, yet there is international consensus that once a TOP is indicated or has been chosen by a woman, the earlier the procedure, the better the clinical and psychological outcomes for the woman. In the UK in 2006, most TOPs (68%) took place before ten weeks gestation and a further 22% took place from 10-12 weeks [9]. In the US in 2001, 59.2% of TOP occurred before the ninth week of gestation, with an additional 19.3% before week ten [2]. In Western Australia in 2004, 71% of TOP had taken place before the ninth week of gestation and 86.6% had occurred by the tenth week [10]. In New Zealand, in 2005 only 17.1% of women who had a TOP did so by week 8 gestation, and only 54.8% by week 10. The remainder 45.2% received TOP services at 11 weeks gestation or later [8]. TOP performed at a later gestational age have implications for the New Zealand health system, not only with respect to an increase in risk of post-procedure clinical complications, but also with respect to emotional repercussions for the women involved. Delays in health care delivery are associated with increased psychological stress. For instance, significant levels of anxiety are present in the course of screening programs, especially in the case of patients who are referred for further testing or who experience delays receiving test results [11]. Long waiting times for surgical procedures also have a negative impact on quality of life and psychosocial measures, including worse general health perceptions and raised levels of anxiety [12]. These negative psychological effects have been found just prior to the anticipated surgery, and are still present up to six months post-procedure [13]. So once a procedure has been committed to, barriers to access and service delivery should be minimized to avoid potential negative psychological complications. Studies investigating the psychological effects of waiting for a TOP procedure have not been conducted in New Zealand, yet women accessing these services are often already under great stress and delay in having the TOP procedure contributes to this stress. Therefore minimizing any delays should be a priority.

This study aimed to establish what the average timeline was for seeking termination of pregnancy services in New Zealand. In particular, it documents average waiting times between different steps of the care pathway, as well as women's perceptions of the timeliness of services.

### TOP Services in New Zealand

TOP services in New Zealand are unique. While they are health services provided mostly in public hospital settings by health professionals, the services, institutions and key personnel are legislated and regulated separately under the Contraception, Sterilisation and Abortion Act and the Abortion Supervisory Committee (AbSC).

**Figure 1:**
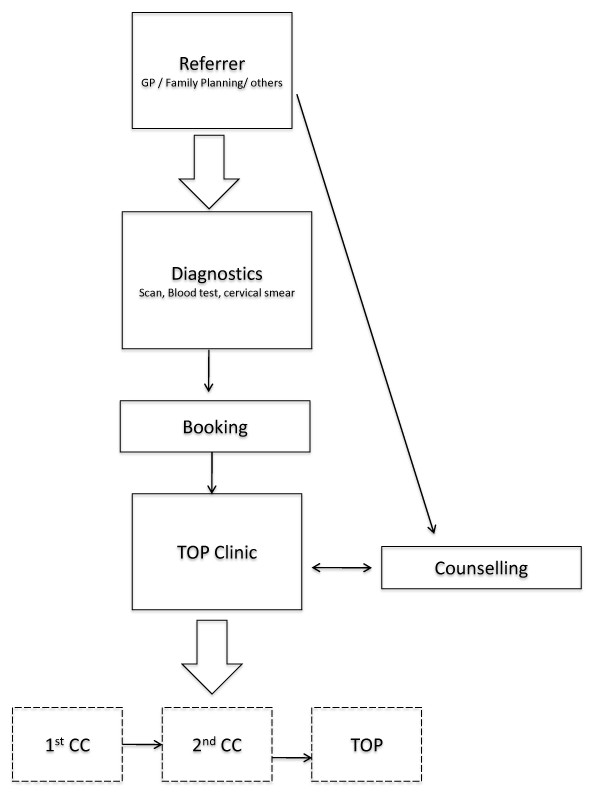
**Termination of pregnancy referral and care pathway in New Zealand**.

Termination of pregnancy is legal under specific circumstances and if conducted by certified practitioners in licensed institutions. The Crimes Act (1961) outlines the circumstances under which pregnancy termination is legal in the country [14]. Pregnancies that present a serious danger to the life of a woman, serious danger to the physical or mental health of a woman, pregnancies resulting from incest or sexual relations with guardian, pregnancies in women of mental subnormality, and pregnancies presenting fetal abnormality may all be legally terminated. Rape and extreme ages are not themselves grounds for termination, but are taken into consideration. The Contraception, Sterilisation, and Abortion Act (1977) outlines the process through which a legal pregnancy termination can be sought [15]. Women who experience an unwanted pregnancy must first go to a referring doctor, usually a General Practitioner (GP) or a Family Planning doctor (FP) for a referral to a TOP clinic. Referring doctors will order diagnostic tests such as blood tests, vaginal swabs, and ultrasound scans, which will then be forwarded to the TOP clinic. Women for whom the decision to terminate or continue with a pregnancy is unclear may also be referred to pre-decision counseling. Most referring physicians will liaise with the TOP clinic to book an appointment for the woman, while some clinics also allow women to telephone and request an appointment themselves. In either case, a written referral from a primary care physician is a legal prerequisite for a termination to proceed. Once at the TOP clinic, women must be seen by two certifying consultants, who are physicians certified to assess the case and attest to the fulfillment of all legal requirements to terminate the pregnancy. Most times, one of the two certifying consultants will also be the operating doctor. By law, all women must be offered counseling throughout the process, although the law does not specify that women must receive counseling. However some clinics around the country have organized their services such that all women attending the clinic receive at least one counseling session, while other clinics continue to offer counseling, but do not require all women to see a social worker or counselor. Figure 1 shows an illustration of the care pathway described.

All New Zealand citizens and residents are entitled to publicly funded TOP services, which are generally provided within the public system. Regions who do not have TOP services available within their district health board purchase TOP services from other regions, including a private clinic in Auckland. Unless they are willing to pay for their own TOP procedure, women generally are unable to choose their TOP provider.

## Methods

A mixed methodology was used to investigate the timeliness of first trimester TOP services in New Zealand. Nine first trimester clinics agreed to participate in this study out of a total of thirteen. Four clinics declined participation for various reasons, such as limited staff and lack of time to take on research. One of the non-participant clinics was a large specialized centre and the remaining three were smaller day clinics within public hospitals. The first methodology used was an audit of the clinical records of all patients attending participating TOP clinics between February 1st and April 30th 2009 (N = 2950). The nine participating clinics ranged in size from 6 beds per week for TOP services to 140 beds per week and together they account for about 70% of all TOPs in the country. Eight clinics were on the North Island and one was on the South Island. In order to extract data from the clinical records at participating clinics, research assistants routinely visited the clinics and recorded the required data in a data collection sheet. All patients were assigned a study identification number in order to protect their identities. In this way, no identifying information, such as name, address or national health identification number, left the clinic premises.

Secondly, participating clinics invited all women attending their clinics during the study period to participate in a patient questionnaire. Women were given a questionnaire to complete in the waiting room and return to a staff member in a sealed envelope. Out of the 2950 women who attended, 1086 agreed to complete a questionnaire (response rate = 36.8%). In order to be able to link the questionnaire information, for those women choosing to complete one, to the audit data, the research team provided study labels to all clinics where the study participant identification number was recorded. Again, this procedure helped ensure that data could be linked while protecting patients' confidentiality. Once outside the clinic premises, no identifiable information was retained.

This study received ethical approval by the Multi-Region Ethics Committee of the Ministry of Health of New Zealand (approval number MEC 08/10/120).

### Variables

At the time of the study, there was no national service guidelines stating the recommended maximum number of days between referral and TOP, as there is in other countries. Throughout this study, delay is defined as the total number of days between each step of the care pathway. For example, the delay between the first contact with the referring doctor and the TOP procedure is the total number of day between the two events. In the case of Medical TOPs, the date of TOP is defined as the date of expulsion of the products of conception. Variables collected through the audit of clinical records included: socio-demographic variables (age, ethnicity, domicile code), parity, gravidity, previous TOPs, date of first contact with referring doctor, date the appointment with the TOP clinic was booked, date of diagnostic tests (ultrasound, blood tests, vaginal swabs), date of counseling appointment, date of first and second certifications, date of termination. When the date of first contact with referrer was not available, the earliest date available from diagnostic tests, referral letter and date when the appointment for the TOP clinics was booked, was taken as proxy. Seven out of the nine clinics had a system in place to record the date that the referring doctor or the patient herself requested an appointment. When this booking date was not available, the earliest date of the date on the referral letter or the date that the referral letter was faxed to the clinic, was taken as proxy.

The patient questionnaire that women were invited to complete included questions about the timing of their decision making process, travel time and transportation partner support, friend and family support, and their perceptions about the timeliness of services.

## Results

Four out of the nine participating clinics provided medical termination of pregnancy (MTOP) as well as Surgical TOPs at the time of the study, and six out of the nine clinics required all patients to see a social worker or counsellor prior to seeing a Certifying Consultant. For the other three clinics, counselling is always offered but entirely optional. Of the 2950 patients seen in participating clinics, 66 (2.2%) had an MTOP. The remaining women had surgical TOPs.

Table 1 shows a description of the audit sample. Women attending TOP clinics during the study period ranged in ages from 14 to 49 years old, with a mean age of 25 (SD = 7.05). Over fifty percent of the sample was New Zealand European. Most women were referred to the service by a GP. Slightly more than 50% of women had had a previous TOP, and 33.4% of the women had had at least two previous TOPs.

**Table 1:** Characteristics of women attending participating clinics


	**Percentages**	**N**

Age		
< 20	28.9	2949
21-25	28.9	
26-30	18.5	
31-35	11.7	
36-40	8.7	
40+	3.3	

Ethnicity		
NZ European	52.5	2950
Maori	22.8	
Pacific	12.3	
Chinese	5.8	
Other Asian	7.5	
Other	11.6	

Type of referrer		
GP	75.8	2936
Family Planning	17.4	
Other	6.3	

Previous TOPs		
0	46.5	2950
1	20.1	
2 or more	33.4	

Gestational age at TOP		
Under 7 weeks 6 days	11.1	2950
8 weeks 0 days - 9 weeks 6 days	34.7	
Over 10 weeks 0 days	54.2	

On average, women underwent their TOP procedure at 70 days gestation (10 weeks, SD = 11.3), and this ranged from 35 days for those having an MTOP to 97 days. Only 11.1% of women had their TOP by the end of the seventh week of pregnancy, and a further 34.7% had their TOP between the eighth week and the end of the ninth week. The majority of women had a TOP procedure during or after the 10^th ^week of pregnancy.

Table 2 shows the average number of days between different steps in the process of seeking a TOP service. Overall, there was a 24.9 days difference between the first contact with the health care system to procure a TOP and the date of the TOP procedure. An average of 10 days went by between first contact and the date the appointment at the TOP clinic was booked, and another 10 days went by between the date the appointment was booked and the first appointment with the TOP clinic. From the first appointment with the TOP clinic to the date of termination, an average of 4 days went by. Table 3 shows the total delay from the first visit with the referring doctor to the date of termination by gestational age at the time of scan. There is a significant relationship between gestational age at scan and delay to termination (using univariate ANOVA, p-value < 0.001). Women who had a scan under 40 days gestation waited for an average of 26.6. Those who had a scan at 41-60 days gestation waited from an average of 25.9 days. Those who had a scan at 61-80 days waited for an average of 20.6 days, and those having a scan at 81 days or more only waited an average of 12.9 days.

**Table 2:** Average number of days delay between different steps of the TOP process


	**N**	**Average number of days**	**Standard** **Deviation**

First contact - booking	2949	10.3	9.1

Booking - counselling	2927	10.6	7.5

Booking - 1^st ^Certifying Consultant	2928	10.5	7.6

Counselling - 1^st ^Certifying Consultant	2402	0.4	3.1

1^st ^Certifying Consultant - 2^nd ^Certifying Consultant	2422	3.2	4.2

2^nd ^Certifying Consultant - TOP	2950	0.9	3.3

First contact - 1^st ^Certifying Consultant	2949	20.8	11.0

First contact - TOP	2950	24.9	10.7

**Table 3:** Average delay by gestational age at the time of scan


**Gestation (days)**	**Delay (days)**	**N**	**SD**

< 40	26.6	471	11.4

41-60	25.9	1913	10.2

61-80	20.6	508	9.8

81+	12.9	53	10.2

Univariate ANOVA p < .0001

All women who attended participating clinics were asked by the clinic staff if they wanted to fill out a questionnaire as part of the study. This sub sample of women was compared to the complete audit sample to see how representative of this sample they were. Women who answered the questionnaire were not significantly different in age from the complete audit sample, but were significantly different in ethnicity. There were a higher percentage of NZ European and Maori women, the two largest ethnicity groups, in the questionnaire sample compared to the audit sample (data not shown).

Women were asked about their decision making process and steps they took before arriving at the TOP clinic (see Table 4). Thirty seven percent of women decided they would have an abortion before or as soon as they thought they were pregnant, and 29.3% decided as soon as they had a positive pregnancy test, while the remaining 34% decided some time after they had a positive pregnancy test. A majority of women saw their referring physician for more than one consultation (64.7%), and the great majority of these return visits were by the referrer's request. Only 15% had to reschedule an appointment due to personal circumstances. Slightly more than 50% of women attended clinics where seeing a counselor or social worker is a non-elective part of the service. Twenty nine percent of women did not see a social worker because they did not want to receive counseling, and surprisingly 3.1% of women reported they were not offered the opportunity to see a social worker or counselor. Most women reported that finding childcare and taking time off work or study was either not difficult or just a bit difficult, but over 50% of women found keeping appointments confidential from others to be moderately to very difficult. During the process of seeking TOP services, most women had the full support of their partner as well as family or friends. Twenty one percent of women stated they did not have a partner and 28% said their friends and family did not know they were pregnant (data not shown).

**Table 4:** Women's decision making and process pre TOP

	Percentages	N
Abortion decision		
Before or as soon as they thought they were pregnant	37.0	1082
As soon as they had a positive pregnancy test	29.3	
Sometime after they had a positive pregnancy test	33.6	

Number of consultations with referring doctor		
1	35.3	1068
2	47.7	
3	13.6	
4 or more	6.5	

Reason for going multiple doctor consultations		
GP asked	81.1	671
I decided to go more than once	18.9	

Patient had to reschedule or change appointment due to personal circumstances	14.8	1069

Social worker seen for counselling		
Yes - I had to	50.7	1079
Yes-I wanted to	14.1	
No-I did not want to	28.6	
No-I was not offered	3.1	
Not yet	3.5	

Patient feels that have had enough counselling support		
Yes	95.4	1033
No	4.6	

Difficulty finding childcare		
Not difficult	37.9	602
A bit difficult	22.7	
Moderately difficult	27.4	
Very difficult	12.0	

Difficulty taking time off from school/work		
Not difficult	29.4	841
A bit difficult	27.9	
Moderately difficult	31.0	
Very difficult	11.7	

Difficulty keeping appointments confidential		
Not difficult	20.8	946
A bit difficult	22.4	
Moderately difficult	35.6	
Very difficult	21.1	

The time it took to organise the abortion was:		
Too long - I wanted an abortion sooner	37.9	1069
Too long - but I didn't mind waiting	15.1	
Just right	39.6	
Quicker than I wanted	2.9	
Other	2.0	
I still don't know when I'm having the abortion	2.6	

Women were asked to estimate their travel time to the TOP clinic. Patients' travel time ranged from 1 minute to 10 hours, and the average travel time to TOP clinics was 55 minutes (SD = 70.8).

When asked what they thought about the time they had waited to get their TOP: 38% thought they had waited too long and would have wanted to get the procedure done sooner, 15% thought it had been too long but they did not mind waiting, almost 40% said the time they waited was just right, and 2.9% thought the timing had been too quick.

Women were then asked to provide feedback on the timeliness of services in an open ended question. Women who thought the timing was too long mostly reported that pregnancy symptoms were severe, interfering with everyday life. Women reported that it proved emotionally difficult to experience symptoms for a pregnancy they would ultimately not keep, and maintaining confidentiality was difficult. On the other hand, women who thought the timing had been long but did not mind waiting commented that they were lucky to be getting the service at all, or that it had given them time to think things through. Women who reported that timing was too quick tended to qualify their answer by stating that it was quicker than they expected, not necessarily quicker than they wanted.

## Conclusions

More than fifty percent of women seeking termination of pregnancy services in participating clinics terminated their pregnancy on or after the tenth week of pregnancy, on average waiting twenty five days between the first time they sought care with a referring doctor until the day of their termination. The longest delays in the care pathway were observed equally between the first contact with the referring doctor and the date of booking for the TOP clinic appointment, as well as between the date of booking and the first appointment with the TOP clinic. Once patients had been seen at the TOP clinic, the remaining steps of the care pathway were completed relatively quickly. Women's' perceptions of the length of time they had to wait reflect these delays, with over half stating that the waiting time was too long. For two thirds of the women who responded to the questionnaire, decision making would not have been a factor delaying them in seeking services; yet gestational age ate scan was significantly associated with delay.

Until recently, there were no guidelines in New Zealand outlining what the acceptable timeline for TOP services was. Taking the guidelines of the Royal College of Obstetricians and Gynaecologists [7] (UK), women on average are waiting almost one week more than the recommended maximum time between the first visit with their GP or referring doctor to the time of the procedure, which is 3 weeks. In October 2009 The Abortion Supervisory Committee published standards of care for women requesting induced abortions in New Zealand [16]. This document defines as a standard that women must not wait longer than two weeks from the time of referral to the time of the TOP procedure, unless women choose to do so to aid their decision making process. Clinic processes around the country are such that there is no single standard referring process, and therefore the date of referral may be defined differently by different clinics. In some instances, clinics will receive telephone requests for appointments before a referral letter is sent, whereas in other clinics only written referrals are accepted as requests for an appointment to be booked. This document has no mention of what timeline should be considered appropriate between the first contact with a referring doctor regarding an unwanted pregnancy, and referral to a TOP clinic. Women who seek referrals early in pregnancy are at particular disadvantage. Currently there is limited availability of medical termination of pregnancy (MTOP), given that not all clinics offer the services and those who do have small lists. Further introduction of MTOP among all clinics may provide these women with the opportunity of terminating a pregnancy without delay.

Future research in this area should focus on the referrers to TOP services. With about half of the total delay attributed to processes before the time of referral and a large proportion of women indicating that their decision to seek a TOP was immediate, health services researchers must understand what factors are at play in slowing down referrals.

There are several limitations to this study that must be acknowledged. This study was conducted during a single three month time period, and therefore is unable to factor in seasonal variation in unwanted pregnancy rates that affect the waiting times in clinics. Secondly, this study collected information at TOP clinics that pertained to care patients received from referring doctors. In many instances, information about the visits with referring doctors was incomplete or missing, and proxy measures were used. Therefore, the delay data should be considered a conservative estimate of the length of time between the first visit with a referring doctor and the TOP procedure. Thirdly, women were able to decline participation in the questionnaire aspect of the study, leading to self-selection bias. It is impossible to discern whether women who experienced particularly long waiting periods were more likely to participate to voice their frustrations, or whether they were less likely to participate. Despite these limitations, this is the first large scale study of TOP services conducted in New Zealand and highlights the need for a closer attention to women's experiences while accessing these services. To avoid further inequities in service, best practices must be identified to ensure that all clinics, regardless of whether within the public or private sector, can minimize the amount of time women have to wait for a procedure.

## Competing interests

The authors declare that they have no competing interests.

## Authors' contributions

All authors participated in the design and development of the study. MS conducted the data analysis and drafted the manuscript. All authors read, edited and approved the final manuscript.
